# Osteopromotion Capacity of Bovine Cortical Membranes in Critical Defects of Rat Calvaria: Histological and Immunohistochemical Analysis

**DOI:** 10.1155/2020/6426702

**Published:** 2020-02-18

**Authors:** Carolina Ferrairo Danieletto-Zanna, Vinícius Ferreira Bizelli, Guilherme André Del Arco Ramires, Tamires Melo Francatti, Paulo Sérgio Perri de Carvalho, Ana Paula Farnezi Bassi

**Affiliations:** ^1^Department of Dentistry, Unicesumar, PhD Student Bauru School of Dentistry, University of São Paulo, São Paulo, Brazil; ^2^Department of Surgery and Integrated Clinic, Araçatuba Dental School, UNESP—São Paulo State University, Araçatuba, São Paulo, Brazil; ^3^Department of Stomatology and Oral Biology—Bauru Dental School, USP—University of São Paulo, Bauru, São Paulo, Brazil

## Abstract

Membranes that aid the guided bone regeneration (GBR) process have been the subject of studies of compatible biomaterials that contribute to this repair process. The present study compared different membranes used in critical-size defects of rat calvaria by assessing GBR as well as histological, histomorphometric, and immunohistochemical reactions. Forty-eight male albino Wistar rats were randomly allocated into four groups (*n* = 12 each), namely, C: membrane-free control group (only blood clot, negative control group); BG: porcine collagen membrane group (Bio-Gide®, positive control group); GD: bovine cortical membrane group (first experimental group); and GDF: thicker bovine cortical membrane group (second experimental group). Rats were euthanized at 30 and 60 days postoperatively. Quantitative data from the histometric analysis were submitted to two-way ANOVA and Tukey's posttest when *p* < 0.05. Histomorphometric results of the thicker bovine cortical membrane at 30 and 60 days were promising, showing improved new bone formation values (*p* < 0.05), and the CD group presented similar results in both analysis periods, being surpassed only by the GDF group (*p* < 0.05). The immunohistochemical results were associated with the histomorphometric data. A less-thick membrane also assisted in GBR. All membranes promoted GBR, especially the positive control and experimental groups.

## 1. Introduction

Insufficient bone volume is one of the main challenges to successful implant rehabilitation [1]. The alveolar bone undergoes vertical and horizontal dimensional alterations after dental extractions, with the reabsorption of the buccal bone plate being more pronounced soon after extraction [2]. In humans, a 50% reduction in the horizontal dimension is evident up to 12 months after the tooth extraction and the most expressive loss occurs in the first 3 months of healing [3].

The increased demand for dental implant treatments in the 1980s spurred the development and refinement of surgical techniques for bone grafting, including alveolar ridge preservation procedures [4], alveolar distraction, and guided bone regeneration (GBR) [5, 6]. GBR is based on the use of resorbable or nonresorbable barrier membranes that prevent the migration of certain types of cells into the bone defect area, such as rapidly growing epithelial cells and connective tissue, favoring the proliferation of osteoprogenitor cells that are able to perform bone neoformation [5, 6].

The membrane used in GBR is an essential component of the treatment process. The membrane must be biocompatible, with a porosity that allows for the diffusion of plasma and nutrients, and must be a barrier to the invasion of epithelial cells [6]. It should also protect the delicate vascular network during clot organization [7], maintain a dimensional stability that supports its weight, and resist the pressure of the adjacent tissues. These attributes prevent the collapse and consequent elimination of the critical space [6, 8, 9].

Barrier biomaterials can be made from a variety of materials [6]. Synthetic polymers, such as expanded polytetrafluoroethylene (e-PTFE), were the first materials used in membranes for GBR [6]. In addition to their capacity to prevent the invasion of connective and epithelial tissue during healing, these biomaterials are sufficiently rigid to create space for osteogenesis [6]. However, they may become exposed to the mouth early, causing contamination of bone graft materials and difficulty in bone regeneration [9, 10].

Seeking to overcome these hurdles, membranes composed of natural polymers were developed [6]. Collagen for these membranes can be derived from different bovine or swine tissues, including the tendon, dermis, pericardium, and small intestine [10], with a thickness of approximately 0.5 to 1 mm for clinical use [11]. The advantages of these membranes include hemostasis, chemotaxis to periodontal ligament fibroblasts and gingival fibroblasts, low immunogenicity, ease of manipulation and adaptation, and ability to increase tissue thickness [10].

Collagen undergoes enzymatic degradation by macrophages and polymorphonuclear leukocytes, which generates carbon dioxide and water [10, 11]. Resorption of the membrane is an important feature. If resorption occurs before bone neoformation, it can cause loss of dimensional stability and dissipation of the bone graft, which consequently impairs GBR. However, delayed reabsorption may be detrimental to wound healing due to the formation of degradation products of a nonfunctional membrane [10].

The type I collagen membrane of bovine cortical bone origin is well tolerated by tissues [12–14]. Complete reabsorption by mono- and multinucleated cells is evident after 30 to 60 days [15]. Recently, a new generation of this membrane became available. The increased thickness of the membrane improves the GBR process.

Several factors might interfere with bone biodynamics and reestablishment of the bone framework for GBR. The objective of this study was to evaluate the osteopromotor potential of three collagen membranes, two membranes of the bovine cortical bone with different thicknesses (GenDerm® and GenDerm Flex®) and a porcine collagen membrane (Bio-Gide®), in the process of GBR following installation in critical bone defects in the calvaria of rats [13–16] at 30 and 60 days using histomorphometric and immunohistochemical analyses.

## 2. Materials and Methods

This study was approved by the Ethics Committee on Animal Experiments at the Araçatuba Dental School, Universidade Estadual Paulista (protocol number 00217-2016), and followed the ARRIVE Guidelines.

Forty-eight male, adult, 3- to 4-month-old rats (*Rattus norvegicus albinus*, Wistar) weighing approximately 200 to 300 g were used. The rats were divided randomly into 4 groups (12 rats per group). They were euthanized 30 days (*n* = 6) or 60 days (*n* = 6) after surgery. The rats were kept in cages, with 3 animals per cage, and fed a balanced diet (NUVILAB, Curitiba, PR, Brazil) containing 1.4% calcium and 0.8% potassium, and they had free access to water at the vivarium of the School of Dentistry, São Paulo State University (UNESP), Araçatuba.

### 2.1. Surgical Procedure

The surgical procedure was performed in the morning at the vivarium of the School of Dentistry, São Paulo State University (UNESP), Araçatuba. The animals were subjected to preoperative fasting for 12 hours before being sedated by intramuscular administration of ketamine hydrochloride (50 mg/kg Francotar; Vibrac do Brasil Ltda, São Paulo, Brazil) combined with xylazine (5 mg/kg Rompun; Bayer S. A. Animal Health, São Paulo, Brazil). Trichotomy was performed in the cranial calvaria region; antisepsis procedures were performed with polyvinylpyrrolidone-iodine (PVPI, 10% Riodeine; Rioquímica, São José do Rio Preto, Brazil) and topical PVPI (10% Riodeine; Rioquímica, São José do Rio Preto, Brazil), and the rats were placed in a sterile field.

**Figure 1:**
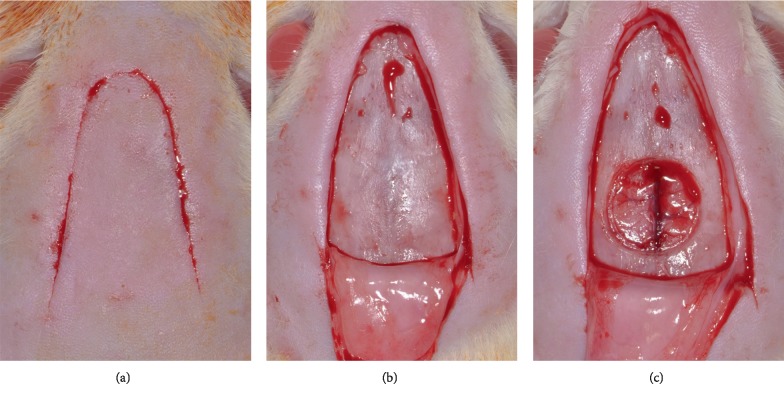
(a) Surgical approach. (b) Total mucoperiosteal detachment. (c) 8 mm diameter bone defect, created in the center of the calvaria by the sagittal suture.

A V-shaped incision of approximately 1 cm on each side was made in the scalp in the anterior region of the calvarium, allowing for reflection of a full-thickness flap in the posterior direction. An 8 mm diameter critical-size defect was made with a 7 mm internal diameter trephine (3i Implant Innovations, Inc., Palm Beach Gardens, USA) housed in a low-speed handpiece with continuous irrigation with sterile saline as previously described. The defect was made in the central portion of the calvaria involving the sagittal suture to maintain the integrity of the dura mater (Figure 1).

Each group was composed of 12 animals. In the C (clot) group, the surgical critical-size defect was filled with a blood clot without overcoating of the defect. In the BG (Bio-Gide®) group, the surgical critical-size defect was filled with a blood clot and was covered by a porcine collagen membrane (Bio-Gide®; Geistlich Pharma AG, Wolhusen, Switzerland). In the GD (GenDerm®) group, the surgical critical-size defect was filled with a blood clot and covered by a thin bovine collagen membrane (GenDerm®; Baumer S. A., Mogi Mirim, Brazil). In the GDF (GenDerm Flex®) group, the surgical critical-size defect was filled with a blood clot and covered by a thicker bovine collagen membrane (GenDerm Flex®; Baumer S. A., Mogi Mirim, Brazil).

After the procedure, the soft tissues were carefully repositioned and sutured at different planes using the resorbable suture thread (polylactic acid, Vicril 4.0; Ethicon, Johnson Prod., São José dos Campos, Brazil) at deep levels, and a monofilament thread (mononylon, Nylon 5.0; Ethicon, Johnson Prod.) with interrupted sutures was used at the most external plane.

In the immediate postoperative period, each animal received a single and intramuscular dose of 0.2 ml of penicillin G benzathine (Veterinary Pentabiotic for Small Animals; Fort Dodge Saúde Animal Ltda., Campinas, SP, Brazil).

The animals were euthanized at 30 and 60 days postoperatively by an overdose of anesthetic (sodium thiopental, 150 mg/kg). The calvaria was removed and set in 10% formaldehyde solution for 48 hours, washed in running water for 24 hours, decalcified in 20% EDTA for 5 weeks, dehydrated in alcoholic solutions, and diaphanized. The prepared calvaria was cut in the middle in the longitudinal direction to separate the bone defects. The obtained pieces were added individually to paraffin, and 6 *μ*m thick sections were obtained. The sections on slides were stained using hematoxylin and eosin.

### 2.2. Morphological Analyses

All morphological analyses were performed using a binocular optical microscope with ×6.3, ×12.5, ×25, and ×40 lenses with an attached AxioCam ICc camera (Carl Zeiss, Oberkochen, Germany) to record images of the tissue sections.

The central region of the surgical wound was analyzed by microscopy. A qualitative analysis was performed by the visual determination of the presence or absence of granulation tissue/young fibrous connective tissue, newly formed blood vessels, fibroblasts, osteoblasts and mineralized bone matrix, foreign body-type granuloma, macrophages, and inflammatory multinucleated giant cells.

### 2.3. Histomorphometric Analyses

For histomorphometric analyses, 12 blades of each experimental group per period (30 and 60 days postoperatively) were assessed. Measurements were performed using an optical microscope (R DMLB; Leica Microsystems Ltd., Heerbrugg, Switzerland) with an attached image capture camera (R DC 300F; Leica Microsystems Ltd.). The images were stored as TIFF files and were analyzed using ImageJ software (National Institutes of Health, Bethesda, MD, USA). The area of bone tissue present in all bone defect extension was assessed. The data obtained from the analyses were transformed into absolute values of pixels to percentages for statistical tests to minimize interference by the negative size difference.

### 2.4. Statistical Analyses

All tests were performed using SigmaPlot 12.3 (Systat Software, Inc., San José, CA, USA). Initially, the data were submitted to the normality test (Shapiro–Wilk), which identified homogeneous data (*p* > 0.05). The two-way ANOVA test was applied for the factors (“membranes” and “periods”) and “membranes × periods” interactions. Additionally, for the accurate identification of the statistical changes, Tukey's posttest was applied. The significance level adopted was 5% for all tests.

### 2.5. Immunohistochemical Analyses

Immunohistochemistry involved the detection of immunoperoxidase activity. Endogenous peroxidase activity was inhibited by hydrogen peroxide. Subsequently, the slides were processed for antigen recovery using phosphate citrate buffer (pH 6.0) and to block endogenous biotin using nonfat dry milk. Primary antibodies were against osteocalcin (Santa Cruz Biotechnology, Dallas, TX, USA) and osteopontin (Santa Cruz Biotechnology). The polyclonal biotinylated secondary goat antibody produced in donkeys (Jackson ImmunoResearch Laboratories, West Grove, PA, USA) was used with an Avidin and Biotin Amplifier Kit (Vector Laboratories, Burlingame, CA, USA). Diaminobenzidine (Dako, Carpinteria, CA, USA) was used as the chromogen. Furthermore, the end of the reaction was carried out against the cut staining with the Harris hematoxylin. For each antibody, the immunolabeling intensity of the relevant proteins was assessed semiquantitatively by assigning different scores, according to the number of cells immunolabeled in the bone repair process. The analysis was performed using the aforementioned R DMLB light microscope. Immunolabeling intensity was scored from 1 to 4, with 1 being the absence of immunostaining and 4 being intense labeling.

## 3. Results

### 3.1. Morphological Analyses

**Figure 2:**
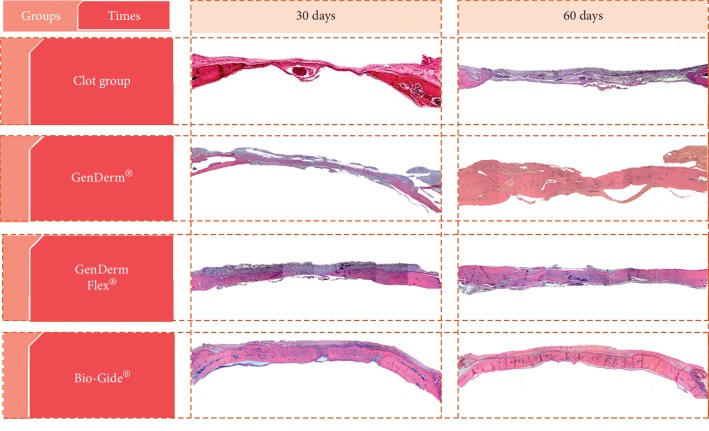
Panoramic histological hematoxylin and eosin-stained sections showing the total area of the groups (C, GD, GDF, and BG). Original magnification, ×6.3.

**Figure 3:**
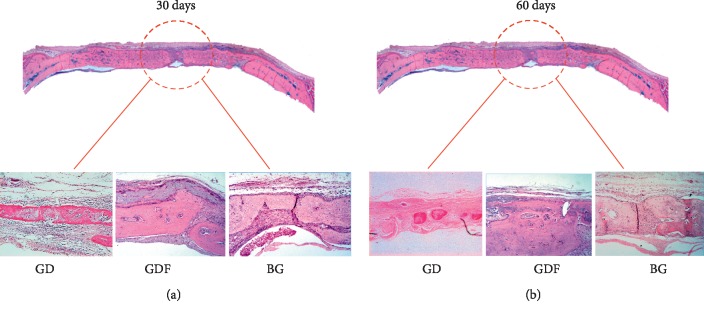
Panoramic histological hematoxylin and eosin-stained section. The images below show the central area of the GD, GDF, and BG groups. Original magnification, ×25 at 30 (a) and 60 days (b).

The results were evaluated by optical microscopy with standardization of the slides from the four groups (Figures 2 and 3).

#### 3.1.1. C Group

- 30 days: an area of major bone neoformation was evident near the border of the defect. The center of the critical defect was filled with loose connective tissue and was not modeled.
- 60 days: closer approximation of the defect stumps was evident without complete closure of the defect. The center of the defect was filled by fibrous connective tissue.

#### 3.1.2. BG Group

- 30 days: a large amount of newly formed bone tissue interspersed by fragments of the Bio-Gide® porcine collagen membrane was observed. In the blades, new bone formation from the bone stumps and in the center of the defect was evident, with the presence of membrane remnants between the area of neoformed bone tissue and the connective tissue organized on the remaining membrane. In some specimens, the defect was closed.
- 60 days: formation of the new bone in the periphery and in the center of the defect was evident, similar to the observations made at 30 days. However, the neoformed bone tissue filled almost the entire cavity. Remnant membrane and well-organized fibrous connective tissue were observed.

#### 3.1.3. GD Group

- 30 days: the defect area was unrepaired, with neoformed bone tissue observed toward the center of the defect. At higher magnification, membrane fragments were observed. Areas of bone neoformation were apparent around the membrane fragments.
- 60 days: new bone formation was detected in much of the defect, but complete closure of the defect was evident in only one specimen. Membrane fragments were rare.

#### 3.1.4. GDF Group

- 30 days: new bone formation was evident from the stump toward the center of the defect. In the periphery of the defect, connective tissue still covered by the membrane was observed. Membrane remnant was observed at the top of every defect. In some rats, the membrane contained organized connective tissue that was well vascularized with many fibroblasts. In others, neoformed bone tissue was noted on the inner face of the membrane. Giant cells were observed adjacent to the outer surface of the membranes. In addition, connective tissue and fibroblasts arranged parallel to the membrane were observed between the membrane and the newly formed bone tissue. Closure of the defect was verified in some specimens.
- 60 days: a large bone neoformation was observed near the bony stump, with the presence of the membrane remnant that was totally involved in bone tissue in its interior and fibrous connective tissue externally. Closure of the center of the defect was evident in most specimens. However, specimens without closure of the defect were also observed. An increased number of giant cells were observed next to the membrane remnant.

### 3.2. Histomorphometric Analyses

**Figure 4:**
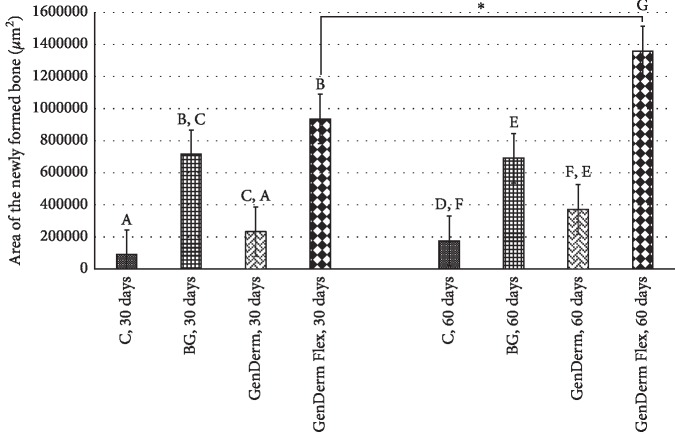
Area of the newly formed bone for the groups at 30 and 60 days. The character *∗* demonstrates statistical intragroup differences (GD and GDF) and the different letters demonstrate statistical intergroup differences (C, GD, GDF, and BG) with Tukey's posttest.

In the histomorphometric evaluation (Figure 4) of the chronological evolution of the bone repair (intragroup analysis), only the time factor demonstrated a statistically significant difference between 30 and 60 days in the GDF group (Tukey's test; *p*=0.021); the other groups did not display statistical differences: GD (Tukey's test; *p*=0.442), BG (Tukey's test; *p*=0.896), and C (Tukey's test; *p*=0.645), but in the GD group, an improvement potential in GBR was observed. In the intergroup analysis, the GDF group demonstrated the best newly formed bone values compared to the positive control group (Tukey's test; *p*=0.01); the groups GD and C did not display a statistical difference (Tukey's test; *p*=0.560).

### 3.3. Immunohistochemical Analyses

**Figure 5:**
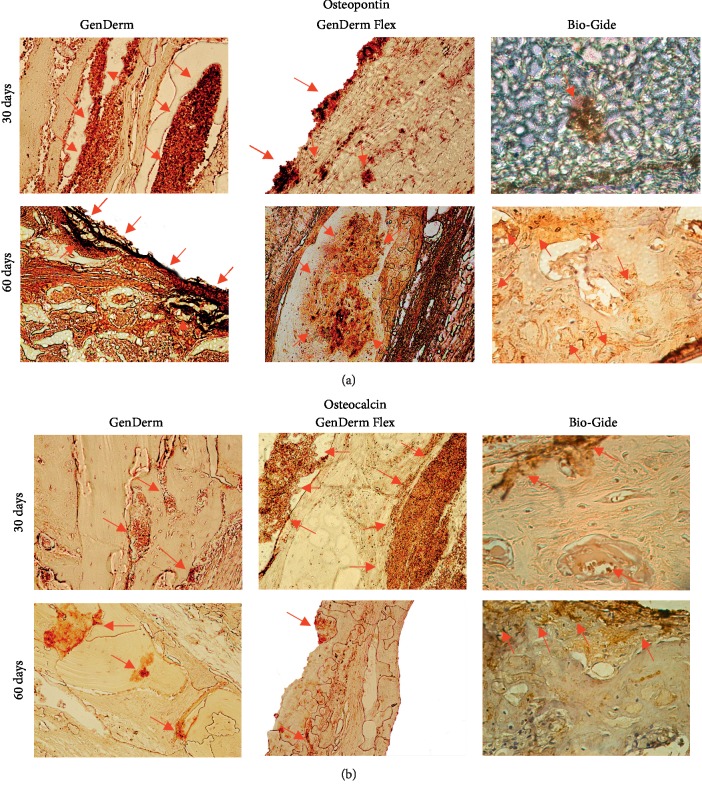
Representative images for osteopontin (a) and osteocalcin (b) immunolabeling at 30 and 60 days postoperatively for the experimental groups and positive control group. Original magnification, ×25.

The results of immunohistochemical analysis are presented in Figure 5 and Table 1 and are detailed below.

#### 3.3.1. BG Group

- Osteopontin: photomicrographs of bone repair at 30 and 60 days revealed light (+) and moderate (++) labeling of the osteopontin protein, respectively.
- Osteocalcin: photomicrographs of bone repair at 30 and 60 days revealed intense marking (+++) of the osteocalcin biomarker in the bone stump region and defect center.

#### 3.3.2. GD Group

- Osteopontin: at 30 days, moderate activity (++) was detected, indicating organization for the repair process. At 60 days, light (+) immunomarking indicated reduced bone matrix formation.
- Osteocalcin: bone repair at 30 days was indicated as moderate-to-intense labeling (++/+++) in the center. The connective tissue was not found to be mineralized in this location. At 60 days, moderate-to-intense labeling (++/+++) of most bone defects was observed.

#### 3.3.3. GDF Group

- Osteopontin: at 30 days, almost all defects were closed and intense labeling (+++) of osteopontin was detected at both the cellular and bone matrix levels. At 60 days, nearly all defects were closed with light labeling of osteopontin (+) once the new bone formation was defined, marking in osteocytes.
- Osteocalcin: intense labeling (+++) was observed at 30 and 60 days.

## 4. Discussion

The primary objective of GBR is consistent and successful bone regeneration in the area of the bone defect with a low risk of complications. Secondly, GBR seeks to obtain a successful outcome with fewer surgical interventions, low morbidity for the patient, and a shortened repair period. In the last 20 years, significant progress has been made in the development of techniques and materials for GBR, with the goal of regeneration in an expected manner [17, 18].

The repair of a control defect (clot without membrane) occurs in a standard manner, with bone formation being restricted to the margins of the defects and the center being filled by fibrous connective tissue. With the introduction of a mechanical barrier in the form of a membrane, there is an increase in the amount of the newly formed bone and tissue regeneration. This occurs because of the space created by the barrier, which separates the endothelium from the bone tissue. The defect that is filled initially by a blood clot undergoes a cicatrization process, which results in greater bone neoformation when compared to the control defect [19, 20].

Critical-size bone defects are considered to have the smallest diameter and do not regenerate spontaneously throughout the life of the animal [21]. Therefore, only biomaterials that are truly capable of assisting in bone healing will be significantly superior to the clot-filled control group [14]. The 8 mm diameter bone defect created in the present study is considered critical according to several studies [13, 14, 21, 22] and the results presented here. The euthanasia time of 30 and 60 days adopted for the experiment was considered the standard in other studies and for representing a period of mineralization and maturation of bone tissue repair in this animal model [23, 24].

Are there differences in the pattern and amount of bone neoformation with the use of different types of membranes? A wide variety of GBR membranes are commercially available. The selection of the material should be based on clinical needs, and the basic properties of the material should include biocompatibility, tissue integration, space formation and maintenance, easy handling, limited susceptibility to complications, and cell occlusion, which avoids invasion of undesirable cells, such as fibroblasts [6, 8, 10].

Collagen membranes are obtained from different bovine or porcine animal tissues (tendon, skin, and intestine) [6, 25]. Although collagen has numerous advantages, such as low immunogenicity, ability to attract gingival fibroblasts, and biocompatibility [10, 25], the rate of degradation is high and collagen may not be maintained for a duration that is sufficient for adequate tissue regeneration [6]. The predictability of the collagen membrane depends on the type of collagen and on various chemical and physical processes to which the collagen must be subjected to eliminate impurities and stabilize its constituent fibrils, which are necessary to improve its mechanical properties and reduce the rate of degradation [6, 10, 25].

The absorbable Bio-Gide® membrane used in the BG group is composed of type I and III pure porcine collagen, with no crosslinking or chemical additives, and is refined to remove antigens [12]. The period of degradation described in the literature varies from 8 weeks [26] to within 4 to 6 months [11, 27]. In the present study, the presence of the membrane at both 30 and 60 days was observed in the morphological analysis, and giant cells were not observed. These observations affirmed that, at 60 days (approximately 8 weeks), the Bio-Gide® membrane showed no signs of degradation and so can be considered a slowly degrading membrane.

The barrier structure consists of two layers. A porous surface is in contact with the bone. A dense surface remains in contact with soft tissues to prevent the growth of fibrous tissue into the bone defect [28]. In the BG group, bone neoformation was observed in the form of islands and the connective tissue was less cellular, suggesting partial bone resorption and penetration of bone tissue. This was probably due to the high permeability of the membrane, which allows for angiogenesis. At 30 and 60 days, more defects were closed, and the bone volume obtained at 30 days seems to be maintained until the end of the repair process at 60 days. Therefore, the membrane used in the BG group maintained a high level of performance in the bone repair process, consistent with previous observations [11, 27].

The membranes used in the GD and GDF groups are composed of type I collagen from the decalcified bovine cortical bone. According to the manufacturer, the GenDerm® membrane thickness is 150–200 *μ*m, which is thinner than that of the GenDerm Flex® membrane, 200–250 *μ*m. The GD group displayed fibrocellular connective tissue, and only fragments of the GenDerm® membrane were detected at 30 and 60 days, with fragments observed less frequently at 60 days. These results corroborate previous studies, which reported membrane fragments [14] or the complete absence of the membrane [13, 16] at 30 days.

In the GDF group, bone neoformation was more progressive. Microscopic analysis revealed slower resorption compared to that in the GD group. In some rats, it was possible to verify the presence of the nearly complete membrane at 60 days. The observation of multinucleated giant cells on the outer surface of membranes in the GDF group at 30 and 60 days suggests the attempted resorption of this biomaterial, as previously reported, which can improve the results of a GBR and provide the surgeon with others options to resolve the difficulties that arise in the everyday clinic [14].

Comparison of the GD and GDF groups revealed a smaller area of bone neoformation in the former group than in the latter, which may be directly related to the faster degradation of the GenDerm® membrane compared to the GenDerm Flex® membrane. The immunohistochemical evaluation revealed a similar biological behavior between the GDF and BG groups, which was confirmed in the comparison of the osteopontin and osteocalcin markers.

Membrane thickness can be analyzed in membranes having the same composition (GenDerm® and GenDerm Flex®). This interfered negatively in the GBR results of the GD group compared to the GDF group, since a thicker membrane resulted in a more effective GBR process compared to the thinner membrane. This can be explained by the early degradation of the membrane in the GD group, which allows for the growth of connective tissue within the defect. This inhibits bone neoformation. The influence of membrane thickness on the temporal maintenance of its integrity has been previously described [13].

Criteria for the choice of membrane that will suit specific clinical issues are based on the results that they demonstrated in studies that have examined their biological behavior. Situations such as alveolar preservations, alveolar ridge augmentation before or at the time of implant placement, and intrabone defects require the use of a membrane, and the GDF group demonstrated that GenDerm Flex® can be a highly viable option in these cases [29].

One of the main limitations of this study was the lack of the available reference material on the use of the GenDerm Flex® membrane. This likely reflects its relatively recent commercialization. The 30- and 60-day analysis periods also limited the evaluation of the complete absorption of the membranes, mainly Bio-Gide®, which at 60 days showed no evidence of the onset of resorption. Studies with longer evaluation periods would be valuable.

## 5. Conclusion

The results support the conclusion that the membranes we examined, Bio-Gide®, GenDerm®, and GenDerm Flex®, promote GBR in critical calvaria defects in rats. The positive control group and the GDF experimental groups displayed better biological behaviors and a higher index of bone neoformation in the GBR process.

## Figures and Tables

**Table 1:** Immunolabeling results at 30 and 60 days.

Groups	OP	OC
BG at 30 days	+	+++
BG at 60 days	++	+++
GD at 30 days	++	++/+++
GD at 60 days	+	++/+++
GDF at 30 days	+++	+++
GDF at 60 days	+	+++

The labeling intensity was represented by the character +, being absent of immunolabeling 0, light +, moderate ++, and intense labeling +++.

## Data Availability

The data used to support the findings of this study are included within the article.

## Supplementary Materials

Supplementary Materials The ARRIVE Guidelines Checklist.
